# Editorial: Atherosclerosis and functional imaging

**DOI:** 10.3389/fcvm.2022.1053100

**Published:** 2022-12-06

**Authors:** Jei-Yie Huang, Yen-Hung Lin, Chung-Lieh Hung, Wen-Pin Chen, Nagara Tamaki, Jeroen J. Bax, Daniel A. Morris, Grigorios Korosoglou, Yen-Wen Wu

**Affiliations:** ^1^Department of Nuclear Medicine, National Taiwan University Hospital, National Taiwan University College of Medicine, Taipei, Taiwan; ^2^Department of Internal Medicine, National Taiwan University Hospital, National Taiwan University College of Medicine, Taipei, Taiwan; ^3^Department of Internal Medicine, MacKay Memorial Hospital, Taipei, Taiwan; ^4^Institute of Biomedical Sciences, Mackay Medical College, New Taipei City, Taiwan; ^5^Institute of Pharmacology, National Taiwan University, Taipei, Taiwan; ^6^Department of Radiology, Kyoto Prefectural University of Medicine, Kyoto, Japan; ^7^Department of Cardiology, Leiden University Medical Center, Leiden, Netherlands; ^8^Department of Internal Medicine and Cardiology, Charité University Hospital, Berlin, Germany; ^9^Department of Cardiology and Vascular Medicine, GRN Hospital Weinheim, Weinheim, Germany; ^10^Department of Nuclear Medicine, Far Eastern Memorial Hospital, New Taipei City, Taiwan; ^11^Division of Cardiology, Cardiovascular Medical Center, Far Eastern Memorial Hospital, New Taipei City, Taiwan; ^12^School of Medicine, National Yang Ming Chiao Tung University, Taipei, Taiwan

**Keywords:** atherosclerosis, functional imaging, cardiovascular, ischemia, plaque, artificial intelligence

Atherosclerosis is a progressive, chronic disorder and the primary cause of cardiovascular diseases such as ischemic heart disease, stroke and peripheral artery disease, which account for ~15 million deaths annually worldwide (1). Most cardiovascular events result from atherosclerotic plaque complications, progression of heart failure and fatal arrhythmia (2–4). Functional imaging has shown potential for the early detection and monitoring of disease progression and therapeutic response, which can ameliorate the burden of cardiovascular disease through the early implementation of interventions or pharmacologic treatment (5). Therefore, this Research Topic focuses on functional imaging for atherosclerosis to evaluate the risk of atherosclerotic plaque and metabolic status of the heart. The studies included in this Research Topic address (1) the development of new molecular imaging tracers, (2) new imaging modalities, (3) new imaging protocols, both for animals and humans, and (4) combinations of both anatomical and functional imaging to improve image quality and diagnostic performance.

Several studies focus on cardiac computed tomography (CT). Cheng et al. demonstrated that extracting quantitative information from conventional images could identify visually imperceptible imaging markers for the precise characterization of vascular morphology and plaques (6). Such an approach can unravel the underlying pathophysiology of atherosclerosis, which bears potential for improved diagnosis, risk stratification and optimal treatment planning. Li et al. evaluated the value of subtraction coronary computed tomography angiography (CCTA) in diagnosing severely calcified coronary artery stenosis in readers with different levels of experience. They prospectively enrolled 47 patients with 134 target coronary segments, and invasive coronary angiography was defined as the reference standard. Their results showed that subtraction CCTA improved the diagnostic accuracy of the radiologists at all experience levels, especially in novice and junior readers. The improvement was most significant in specificity, with an increase from around 30% to 80%. In addition, using invasive coronary angiography with additional fractional flow reserve (FFR) as the reference standard, Yan et al. and Gao et al. evaluated the diagnostic performance of CT-derived FFR (CT-FFR). Yan et al. evaluated the diagnostic performance of CCTA, CT-derived FFR (CT-FFR) and change in CT-FFR across the lesion (ΔCT-FFR) to identify stenosis at a lesion-based level. After retrospectively analyzing 152 patients, they concluded that CT-FFR and ΔCT-FFR improved the diagnostic performance compared with CCTA alone. They suggested that ΔCT-FFR reflected the change in CT-FFR proximal and distal to the lesion, and that it demonstrated the change in hemodynamics of the lesion directly. Further, they suggested that this resulted in better diagnostic performance than CT-FFR, which was measured distal to the vessel and tended to overestimate ischemic lesions. Yan et al. also investigated CT-derived plaque characteristics to predict ΔCT-FFR, and found that low-attenuation plaque volume and plaque length were independent risk factors. Gao et al. performed a prospective multicenter clinical trial (NCT03692936) which also evaluated the diagnostic performance of CT-FFR obtained by a new computational fluid dynamics (CFD) algorithm. They analyzed 317 patients, and concluded that CT-FFR based on the new parameter-optimized CFD model provided better diagnostic performance than CTA, especially in improving the diagnostic accuracy of “gray zone” lesions. They also showed that Agatston score increased the false-negative rate, and that coronary stenosis >50% increased the false-positive rate for the detection of myocardial ischemia.

Even though FFR can be used to pathophysiologically evaluate the functional significance of coronary lumen narrowing, optical coherence tomography (OCT) allows for precise visualization and quantification of high-risk plaque features (7, 8). However, OCT requires the use of a large amount of contrast media, which may limit its clinical use. Chen et al. compared OCT images of coronary lesions obtained using contrast media and a very-low amount of contrast media combined with Ringer's solution (VLCCR) in 30 patients with acute coronary syndrome. They concluded that VLCCR for blood clearance was feasible and safe, and that it provided similar image quality to that obtained using contrast media. Recently, dynamic computed tomography myocardial perfusion imaging (CTP) has been developed and shown to be a useful adjunct to CCTA for diagnosing myocardial ischemia, and to provide quantitative perfusion parameter data (9). Geng et al. investigated the feasibility of reducing radiation exposure in a swine model by reducing the tube voltage. Seven swine underwent rest and stress dynamic CTP with tube voltages of 100 and 70 kV, and the results showed that 70 kV yielded an ~64.6% radiation dose reduction while generating comparable myocardial blood flow values, both at rest and stress states. Reducing the radiation dose may promote the clinical use of CTP. Combining OCT and CTP may improve the evaluation of plaque characteristics and cardiac ischemia, although increased radiation exposure is still a concern.

Speckle-tracking is an advanced quantitative echocardiographic technique which can assess myocardial function, deformation, ventricular systolic and diastolic dynamics (10). Sung et al. investigated associations among sex, menopause and duration of menopause on cardiac geometry and mechanics using a chamber-specific, speckle-tracking technique. They showed that greater left ventricular sphericity, impaired global longitudinal strain, reduced peak left atrial longitudinal strain and higher left atrial stiffness were independently associated with heart failure hospitalizations in postmenopausal women. The prognostic value of these parameters could partly explain the influence of sex in heart failure. Further studies to validate and explore the underlying mechanisms may reduce the gap in knowledge for the management of heart failure in certain patient subgroups.

Artificial intelligence techniques are increasingly being used in data science. In cardiovascular imaging, problems with timing, efficiency, and missed diagnoses may occur at all stages. The application of artificial intelligence may reduce these problems and improve all stages of image acquisition, interpretation, and decision-making (11, 12). The strength of machine learning is that it can evaluate a great amount of information and find significance. Lin et al. used machine learning analysis of carotid sonographic features to predict recurrent stroke. They enrolled 2,411 patients and analyzed a total of 1,235 carotid sonographic parameters, and concluded that CatBoost was an optimal model to predict recurrent stroke. In addition, the top three features were the use of anticoagulation, non-steroidal anti-inflammatory drugs, and resistive index of the left subclavian artery. Furthermore, deep learning algorithms can also be used to identify imaging features and patterns, and they have been shown to be able to predict obstructive coronary artery disease (13).

From an imaging perspective, positron emission tomography (PET) is a leading tool for imaging molecular processes because of its high spatial resolution, high sensitivity and advantages in visualizing molecular processes in living human tissue (14). The pathogenesis of atherosclerosis includes a cascade of molecular events (15), and imaging can be used to assess each step. Imaging atherosclerosis with new and novel PET radiotracers and evaluating its association with stroke and multi-vessel coronary artery disease can help elucidate the basic mechanisms of atherosclerosis (16–20). In addition, the reproducibility and *in vivo* imaging can be useful in the longitudinal follow-up of disease status (21). Another increasingly popular imaging modality, cardiovascular magnetic resonance (CMR), provides comprehensive information without the need for radiation, and it has recently been used to evaluate the etiology and predict the outcomes of cardiovascular diseases (22, 23). In addition, the recently introduced PET-magnetic resonance imaging (MRI) has been shown to have a key role in identifying high-risk coronary artery disease using [^18^F] fluoride PET and gadobutrol-enhanced MRI (24). No studies on PET or CMR have been included in this Research Topic, however we anticipate investigations on their application to atherosclerosis in the future.

In conclusion, this Research Topic broadens the knowledge of atherosclerosis with regards to molecular mechanisms, as well as the diagnostic and prognostic value of different imaging modalities. A multimodality approach may enhance the clinical application of cardiovascular imaging to characterize atherosclerotic disease in the future, allowing not only for a better understanding of the underlying pathophysiology but also for the development of more efficient treatment strategies in the field of cardiovascular medicine.

## Author contributions

All authors listed have made a substantial, direct, and intellectual contribution to the work and approved it for publication.
